# Relationship between the anti-inflammatory properties of salmeterol/fluticasone and the expression of CD4^+^CD25^+^Foxp3^+ ^regulatory T cells in COPD

**DOI:** 10.1186/1465-9921-12-142

**Published:** 2011-10-28

**Authors:** Li Yang, Qian-li Ma, Wei Yao, Qiao Zhang, Hua-ping Chen, Guan-song Wang, Chang-zheng Wang

**Affiliations:** 1 Institute of Respiratory Diseases, the Second Hospital of the Third Military Medical University of China, 183 Xinqiao Street, Chongqing 400037, P. R. China

**Keywords:** T-lymphocytes, inflammatory mediators, chronic obstructive pulmonary disease, salmeterol and fluticasone propionate

## Abstract

**Background:**

Salmeterol and fluticasone combination (SFC) has anti-inflammatory effects and improves clinical symptoms in patients with chronic obstructive pulmonary disease (COPD). However, the anti-inflammatory mechanism of SFC remains unclear. In this study, we investigated the inflammatory responses of COPD, as well as the relationship of the inflammatory factors with the levels of CD4^+^CD25^+^Foxp3^+ ^regulatory T cells (Foxp3^+^Tregs) after SFC therapy.

**Methods:**

Twenty-one patients with moderate or severe COPD received treatment with 50/500 μg of SFC twice a day for 12 weeks. Before and after treatment, the patients were evaluated using the Modified Medical Research Council (MMRC) dyspnea scale and by conducting a 6-min walk test. The number of neutrophils, monocytes and lymphocytes in induced sputum were counted. Levels of cytokines, including pre-inflammatory IL-8, TNF-α, IL-17A and cytokine IL-10, in the sputum supernatant and peripheral blood were measured by ELISA. The proportion of Foxp3^+^Tregs in the total CD4^+ ^T cell of the peripheral blood was determined by flow cytometry. The relationship between IL-17A levels and the percentage of Foxp3^+^Tregs was analyzed by statistical analysis.

**Results:**

After treatment with SFC, the forced expiratory volume in 1 s as a percentage of predicted values (FEV1%) and the 6-min walk distance in the COPD patients significantly increased, while dyspnea scores decreased. The total number of cells, neutrophils, and the percentage of neutrophils in induced sputum reduced notably, while the proportion of monocytes was significantly increased. Levels of the inflammatory cytokines IL-8, TNF-α, and IL-17A in the sputum supernatant and in the blood were markedly lowered, while IL-10 levels were unchanged. The proportion of Foxp3^+^Tregs in the total CD4^+^T cell population in the peripheral blood was drastically higher than that before treatment. The level of IL-17A was negatively correlated with the proportion of Foxp3^+^Tregs in CD4^+^T cells.

**Conclusion:**

SFC can reduce the levels of inflammatory factors and improve symptoms of COPD. The levels of inflammatory factors are associated with the variation of Foxp3^+^Tregs in COPD.

**Trial registration**

This study was registered with http://www.chictr.org (Chinese Clinical Trial Register) as follows: ChiCTR-TNC-10001270

## Introduction

Chronic obstructive pulmonary disease (COPD) is a chronic disorder characterized by persistent inflammation of the lung [1,2], which is mainly caused by cigarette smoking and inhalation of polluted gas or particles. Tobacco smoking is considered the primary risk factor for COPD [3]. Although the pathophysiological progression of COPD can be delayed by quitting smoking, the inflammatory reactions will continue and is irreversible [4-6].

Recent reports indicate that the acquired immune response is involved in the pathogenesis of COPD [7]. This could account for the persistence of inflammation in the lungs after COPD patients stop smoking. First, epithelial cells damaged by smoking release chemokines and cytokines to activate and stimulate chemotaxis of neutrophils and alveolar macrophages, which initiate innate inflammation. This process also leads to maturation and migration of dendritic cells into pulmonary lymphoid tissues, the secretion of inflammatory cytokines, and CD4^+^/CD8^+ ^T cell differentiation and migration into the lung. Immune regulation and immune tolerance determine the immune response to and outcome of COPD. If the dendritic cells lose immune tolerance to autoantigens from inflammation-injured lung tissue, they will activate type 1 helper T cells (Th1) and cytotoxic CD8^+ ^T cells, and trigger the acquired immune response and inflammation [4]. CD4^+^CD25^+^Foxp3^+ ^regulatory T cells (Foxp3^+^Tregs) play an important role in the regulation of these processes [8]. Reduction in the number of Tregs weakens immune tolerance to autoantigens [9]. The number of Tregs in the lungs of smokers without lung dysfunction is more than that in people without a history of smoking [4]. Tregs may be involved in the maintenance of normal lung function in smokers and may stop the progression of immune responses [10]. Furthermore, Foxp3^+^Tregs could improve prognoses in cases of acute exacerbation of COPD [11].

Acquired immune response-related cytokines and inflammatory mediators actively participate in the pathogenesis of COPD. A better understanding of the acquired immune response and T cells will lead to improved intervention strategies in cases of COPD and will help reduce airway inflammation. A variety of treatments can relieve the symptoms and improve the quality of life of COPD patients, but these treatments cannot control inflammation or block the progression of COPD [12]. Inhaled long-acting bronchodilators alleviate symptoms to some extent and improve patients' lung function and quality of life. Corticosteroids inhalation can avoid acute exacerbation of patients' symptoms and reduce frequent deterioration of a patient's condition. A combination of bronchodilators, salmeterol, and a corticosteroid, or fluticasone propionate (SFC) has better efficacy than either drug has [13-15]. SFC has been shown to have anti-inflammatory effects in patients with COPD [16], which contributed to an improvement in symptoms. However, whether the clinical efficacy of SFC is mediated by Foxp3^+^Tregs in peripheral blood is unknown. In this study, we hypothesized that SFC alleviated inflammation in patients with COPD by increasing the percentage of Foxp3^+^Tregs in the peripheral blood to enhance immune tolerance, thereby reducing the total numbers of cells and neutrophils in induced sputum, and reducing the levels of inflammatory factors such as IL-8, TNF-α, and IL-17A.

## Methods

### Subjects

A total of 21 subjects with a clinical diagnosis of stable COPD [1] at or above mid range were enrolled between September 2009 and January 2010 from outpatient clinics at the Xinqiao Hospital, Chongqing, China. The eligibility criteria for the study were as follows: age between 40 and 79 years, habit of smoking or a history of smoking (≥ 10 pack-years), forced expiratory volume in 1 s (FEV1) after administration of a bronchodilator < 80% of the predicted value, FEV1/forced vital capacity (FVC) < 70% of the predicted value, and no history of asthma, atopy (as defined by a positive reaction to one or more allergen in a fluoro- enzyme immunoassay) or any other active lung disease. Subjects were either newly diagnosed or had not received corticosteroids (either oral or inhaled), antibiotics, or any other long-acting β2-agonists for a minimum of 30 days prior to the commencement of the study. All subjects had been free from respiratory tract infections or COPD exacerbation for 12 weeks prior to the pulmonary function tests and sputum induction. Eleven subjects with a smoking history (≥ 10 pack-years) and normal lung function were used as healthy control. The study protocol was approved by the ethics review board of the Second Affiliated Hospital, Third Military Medical University, China. Written informed consent was obtained from all participants, and all of the procedures were done in accordance with the Declaration of Helsinki and relevant policies in China. The protocol was also proved by Chinese Clinical Trial Register (Chengdu, China). Trial registration number: ChiCTR-TNC-10001270.

### Study design

**Figure 1:**
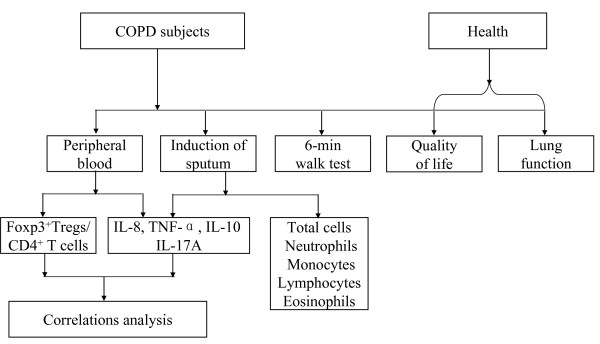
**Schematic diagram for study design**.

The study was an own anterior-posterior control clinical trial, with the subjects allocated to receive treatment as follows: SFC 50/500 μg per puff, 2 puffs twice daily by using an Evohaler (GlaxoSmithKline, Ware, UK). Use of theophylline and any other short-acting β2-agonists was discontinued during the study. The treatment duration was 12 weeks, with clinical visits at weeks -2, 0, 4, 8, and 12. Peripheral blood was collected from healthy control and test subjects. The percentage of Foxp3^+ ^Tregs as a proportion of the total CD4^+^T cells in the blood was tested by flow cytometry. The peripheral blood samples were stored at -70°C, and the levels of IL-8, TNF-α, IL-10, and IL-17A in the blood were subsequently tested using ELISA (R&D Systems, Abingdon, UK) according to the manufacturer's instructions. Induced sputum was processed as described previously [17,18], and the supernatant was aspirated and frozen at -70°C prior to the measurement of inflammatory mediators. The levels of inflammatory mediators, including IL-8, TNF-α, IL-10, and IL-17A, in the supernatants were assayed using ELISA. Pulmonary function tests, sputum induction, the 6-min walk distance [19], and assessment of health-related quality of life were carried out in the morning at screening and in week 0 and 12. Health status was assessed using the MMRC scale (Figure 1).

### Peripheral blood collection and processing

The peripheral blood samples were collected in heparin-treated tubes from each subject in week 0 and 12. Eight milliliters of peripheral blood were drawn simultaneously. Four milliliters of peripheral blood was isolated for lymphocytes. Surplus were centrifuged at 1,200 g for 10 min. The cell-free supernatants of serum were frozen at -70°C for determining cytokine concentrations.

### Flow cytometry and FACS analysis

The expression markers on T cells from blood were determined by flow cytometry after surface staining or intracellular staining with anti-CD4, anti-CD25, and anti-Foxp3 mAbs Human Regulatory T cell Staining Kit (eBioscience, Inc, San Diego, USA). Peripheral blood mononuclear cells (PBMCs) were isolated from whole blood on a discontinuous Histopaque density gradient (Sigma). The extracellular cells were stained with fluorescein isothiocyanate (FITC) conjugated anti-human CD4, allophycocyanin (APC) conjugated anti-human CD25 (eBioscience, Inc, San Diego, USA). Lymphocytes were gated in FSC and SSC stained. The percentage of CD4^+ ^cell type was counted out of gated CD4^+ ^cells in lymphocytes. CD25^+ ^cells were expressed on the gated CD4 cells. Intracellular staining of phycoerytrin (PE) conjugated anti-human Foxp3 was conducted according to the recommended procedure (eBioscience, Inc, San Diego, USA). Each sample was added freshly eBioscience Foxp3 fixation/permeablization buffer and incubated at 4°C for 60 minutes. cells were washed and centrifuged at 4°C for 10 minutes, 300 g. Anti-human Foxp3 antibodies were used in the same test tube. The percentage Foxp3^+ ^was determined out of gated CD4^+ ^lymphocytes. Flow cytometry data acquisition was performed on a FACS Calibur equipped with 488 and 633 nm lasers and running Cellquest Software (Becton Dickinson, CA, USA). Fifty thousand live-gated events were collected for each sample and isotype matched antibodies were used to determine binding specificity. Flowjo software was used for data analysis (Treestar, OR, USA).

### Sputum induction and processing

Sputum was induced as reported by Pin et al. [17]. In short, a 3% hypertonic saline solution was nebulized via anultrasonic nebulizer and inhaled by the subjects. The sputum collected from each subject was pooled and kept at 4°C for not more than 2 hours prior to further processing. The sputum was stained with Giemsa stain and then processed with dithiothreitol at a final concentration of 0.1%, as has been described. Sputum was diluted and centrifuged (1,500 g, 4°C, 10 min) and then the supernatants were stored at -70°C for determining cytokine concentrations. Differential cell counts were expressed as the percentage of non-squamous cells [18]. Absolute cell numbers were calculated as (% cell × total cell count)/(sputum weight). The percentage of squamous cell < 20% were qualified.

### Enzyme-linked immunosorbent assay (ELISA)

The concentrations of IL-8, TNF-α, IL-10, and IL-17A in the supernatant of the peripheral blood and induced sputum were measured by sandwich ELISA kits according to the manufacturer's protocols (R&D Systems, Abingdon, UK). The lower detection limits of IL-8, TNF-α, IL-10, and IL-17A were 62 pg/ml, 20 pg/ml, 12.5 pg/ml, and 62.5 pg/ml, respectively.

### Pulmonary function test

Dynamic spirometry (FVC and FEV_1_) was performed pre- and post- bronchodilatation using a Vitalograph Spirometer (Jaeger, Würzburg, Germany) according to the guidelines of the American Thoracic Society.

### Six-min walk distance and MMRC scale

COPD patients were requested to walk a 30-m course in a flat corridor back and forth for 6 minutes (6-min walk test). Patients may rest if they were too short of breath to continue after adequate encouragement.

The degree of dyspnea was measured with the use of the modified Medical Research Council (MMRC) dyspnea scale. The scores range from 0 to 4, with a score of 4 indicating that the patient is breathless when dressing or undressing [19].

### Statistical analysis

Statistical analysis was performed using the SPSS 15.0 statistical package. Data are expressed as mean ± SEM. For multiple comparisons, statistical analysis was performed using ANOVA, and the post hoc test was subsequently applied as the least significant difference test for pairwise comparisons. Association in treatment differences (pre-treatment minus post-treatment) between the levels of IL-17A and the proportion of Foxp3Tregs, as a percentage of total CD4^+^T cells, was measured using Spearman's rank correlation test. A p value of 0.05 was considered significant for all tests.

## Results

### Subject characteristics

All of the 21 patients with COPD (20 men and 1 woman, aged 40-79 years, mean 66 years) had a history of smoking. Eight patients had moderate COPD, and 13 had severe COPD. The smoking index ranged from 12.5 to 89 packs per year, with an average of 36 packs per year. The FEV1 percentage after treatment (post-FEV1%) was 27% to 65% (mean 45.62 ± 12.06%). The mean FEV1 value was 1.16 ± 0.38 L (Table 1). None of the patients experienced exacerbation of symptoms during the study period.

**Table 1:** Characteristics of COPD patients and health

	COPD	Health
Subjects (n)	21	11
Males (n)	20	9
Females (n)	1	2
Mean age (years)	66.19 ± 8.43	63.55 ± 6.58
Duration of disease (years)	12.95 ± 9.17	-
Smoking history (pack-years)	36.45 ± 20.18	-
Mean FEV1(L)	1.16 ± 0.38	2.45 ± 0.69
Mean FEV1%, predicted	45.62 ± 12.06	103.82 ± 17.05
Post-FEV1%, > 80% (n)	0	11
Post-FEV1% 50%-80% (n)	8	0
Post-FEV1% 30%-50% (n)	13	0
Mean FVC (L)	3.19 ± 0.86	3.21 ± 0.83
Mean FEV1/FVC(%)	36.76 ± 9.27	76.18 ± 4.47
Mean reversibility (%)	14.05 ± 13.33	-
Mean reversibility (L)	0.15 ± 0.13	-

Data are presented as mean ± SD.

FEV1, forced expiratory volume in 1 s. FVC, forced vital capacity.

### Effect of SFC therapy on lung function and quality of life in COPD patients

In the cases of patients treated with SFC for 12 weeks without bronchodilators, FEV1% values increased significantly (p = 0.016) from 39.62 ± 2.35% (before therapy) to 46.10 ± 3.58% (pre-FEV1%). Administration of bronchodilators further increased FEV1% to 49.00 ± 3.22% (post-FEV1%), but this change did not reach statistical significance (p = 0.100) relative to the values before treatment (45.62 ± 2.63%). The pre-FEV1 values and post-FEV1 values also showed no significant difference before (1.02 ± 0.07 L and 1.16 ± 0.08 L, respectively) and after SFC therapy (1.15 ± 0.13 L and 1.27 ± 0.11 L, respectively). The value of mean reversibility significantly decreased (p = 0.048) from 0.15 ± 0.03 L to 0.08 ± 0.02 L, but the decrease in percentage mean reversibility (14.05 ± 2.91% before therapy vs. 8.19 ± 1.96% after therapy) was not significant. Regarding clinical parameters, after the combination therapy, dyspnea measurements were significantly lower (2.24 ± 0.01 before vs. 1.33 ± 0.11 after treatment) (p < 0.001), and the 6-minute walk distance was significantly greater (329.52 ± 8.82 m before vs. 367.14 ± 7.99 m after therapy) (p < 0.001) (Table 2). These data show that the quality of life of the COPD subjects improved after SFC therapy.

**Table 2:** Effect of SFC therapy on lung function and quality of life in COPD patients

	Baseline	SFC therapy
Pre-FEV1 (%)	39.62 ± 2.35	46.10 ± 3.58*
Pre-FEV1 (L)	1.02 ± 0.07	1.15 ± 0.13
Post-FEV1(%)	45.62 ± 2.63	49.00 ± 3.22
Post-FEV1 (L)	1.16 ± 0.08	1.27 ± 0.11
FVC (L)	3.19 ± 0.19	3.25 ± 0.22
FEV1/FVC (%)	36.76 ± 2.02	39.71 ± 2.65
Mean reversibility (%) Mean reversibility (L)	14.05 ± 2.91 0.15 ± 0.03	8.19 ± 1.96 0.08 ± 0.02 *
MMRC dyspnea scale	2.24 ± 0.10	1.33 ± 0.11 ***
6-min walk test (m)	329.52 ± 8.82	367.14 ± 7.99 ***

* p < 0.05, *** P < 0.001

### Effect of SFC therapy on the induced sputum of moderate and severe COPD patients

**Figure 2:**
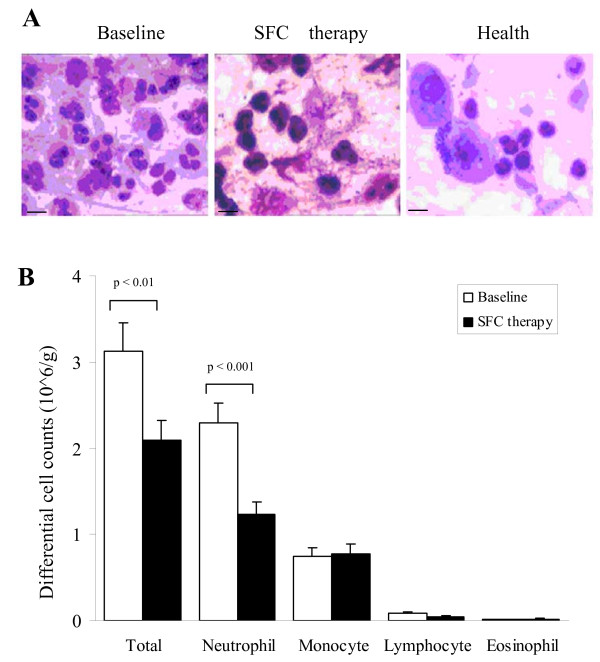
**Total cell number and neutrophils decreased in induced sputum of COPD patients after SFC therapy**. (A) Photomicrograph of Giemsa staining on sputum cells of COPD patients in Baseline (left) and SFC therapy (middle), or healthy controls (right). 20 μM. (B) Histogram shows the numbers of total and the inflammatory cells, including neutrophils, monocytes, lymphocytes and eosinophils in the induced sputum of COPD patients. p < 0.01, p < 0.001, compared with SFC therapy.

After SFC therapy for 12 weeks, the total number of cells and neutrophils in the induced sputum significantly decreased from 3.13 × 10^6 ^cells/g to 2.09 × 10^6 ^cells/g (p = 0.002), and from 2.29 × 10^6 ^cells/g to 1.24 × 10^6 ^cells/g (p < 0.001) as compared with baseline (Figure 2). While monocyte numbers in the induced sputum kept little change (from 0.74 × 10^6 ^cells/g to 0.78 × 10^6 ^cells/g, p = 0.76, Figure 2). The similar with these tendencies, the percentage of neutrophils and monocytes decreased by 14.26% (p = 0.001) and 13.81% (p = 0.001) as compared with baseline, respectively. Indeed, differences in the absolute number and percentages of lymphocytes and eosinophils before and after SFC treatment were not significant, respectively (p > 0.05, Figure 2). These results suggest that the number of total and neutrophils reduce and neutrophil-related inflammation in the airways is ameliorated after SFC therapy.

The distinction between moderate and severe COPD patients in the induced sputum was compared with each other in statistical methods. The results showed that, for severe COPD patients, total cell numbers and neutrophil numbers marked decreased from 3.15 ± 0.44 to 2.05 ± 0.27 (× 10^6^/g), 2.35 ± 0.31 to 1.25 ± 0.18 (× 10^6^/g) after SFC therapy (p = 0.02, p = 0.001, Additional file 1*Table S1*). For moderate COPD patients, neutrophil numbers much reduced from 2.19 ± 0.34 to 1.23 ± 0.24 (× 10^6^/g) after SFC treatment (p = 0.02, Additional file 1*Table S1*). There are no difference about the monocyte numbers in moderate and severe COPD between before and after SFC therapy (p = 0.90, p = 0.75). And there are no difference about total cell numbers and the percentage of neutrophils and monocytes in moderate COPD patients after SFC therapy (p = 0.08, p = 0.10, p = 0.12). There are no difference about the lymphocytes and eosinophils in the induced sputum, neither in moderate nor severe COPD (Additional file 1*Table S1*). These data suggest that there is an obviously change about inflammatory cells in total and severe COPD patients at present subjects.

### Effect of SFC therapy on the levels of inflammatory mediators in induced sputum and serum

Levels of the inflammatory factors IL-8, TNF-α, and IL-17A in the supernatant of induced sputum decreased significantly to 25.69 ± 3.50 ng/ml (p < 0.001), 562.79 ± 29.76 pg/ml (p < 0.001), and 0.33 ± 0.03 ng/ml (p = 0.025), respectively, after SFC therapy (Table 3). The increase in IL-10 level in the sputum after treatment was not statistically significant (p = 0.121). After treatment, the levels of IL-8, TNF-α, and IL-17A in serum also decreased significantly to 7.53 ± 0.40 ng/ml, 148.97 ± 14.33 pg/ml, and 0.95 ± 0.06 ng/ml, respectively (p < 0.001, p = 0.008, p = 0.022, respectively, Table 4). The increase in the serum levels of IL-10 was not significant (p = 0.785, Tables 3 and 4). These data showed that the levels of inflammatory factors in COPD subjects decreased after SFC therapy.

**Table 3:** Effect of SFC therapy on the levels of cytokines in the induced sputum of COPD

	Baseline	SFC therapy
IL-17A (ng/ml)	0.40 ± 0.02	0.33 ± 0.03*
TNF-α (pg/ml)	802.71 ± 42.63	562.79 ± 29.76 ***
IL-8 (ng/ml)	43.09 ± 4.63	25.69 ± 3.50 ***
IL-10 (pg/ml)	39.09 ± 3.75	44.94 ± 4.92

* p < 0.05, *** p < 0.001

**Table 4:** Effect of SFC therapy on the levels of cytokines in the blood of COPD

	Baseline	SFC therapy
IL-17A (ng/ml)	1.06 ± 0.06	0.95 ± 0.06*
TNF-α (pg/ml)	212.88 ± 13.10	148.97 ± 14.33**
IL-8 (ng/ml)	9.97 ± 0.43	7.53 ± 0.40 ***
IL-10 (pg/ml)	47.43 ± 7.71	50.59 ± 6.17

* p < 0.05, ** p < 0.01, *** p < 0.001

### Effect of SFC therapy on the expression of CD4^+^CD25^+^Foxp3^+ ^Treg cells

**Figure 3:**
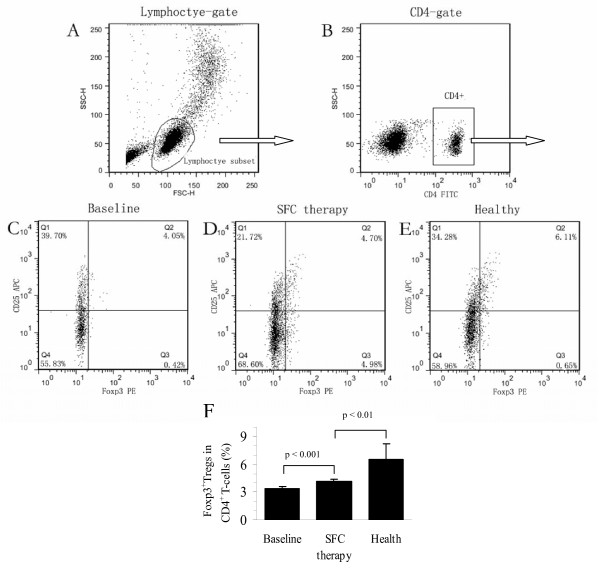
**CD4^+^CD25^+^Foxp3^+ ^Tregs increased in the peripheral blood of COPD by SFC therapy**. Flow cytometry analysis of Foxp3^+^Tregs in COPD patients before and after SFC treatment. (A) Lymphocytes (R1) were identified based on their characteristic properties shown in the forward scatter (FSC) and sideward scatter (SSC). (B) A representative gating was set for CD4^+ ^T cells from blood lymphocytes (R2). (C) A representative dot plots showing expression of CD25^+^Foxp3^+ ^Tregs in blood CD4^+ ^T cells of COPD before SFC therapy (Baseline). (D) In COPD after SFC therapy, a representative dot plots showing expression of CD25^+^Foxp3^+ ^Tregs in blood CD4^+ ^T cells. (E) In healthy controls, a representative dot plots showing expression of CD25^+^Foxp3^+ ^Tregs in blood CD4^+ ^T cells. (F) Comparison with the percentages of CD25^+^Foxp3^+^Tregs (Foxp3^+^Tregs) in COPD blood before and after SFC treatment by histogram. p < 0.001, compared with that in COPD patients before SFC therapy. p < 0.01, compared with that in healthy controls.

Since Foxp3^+^Tregs have been shown to participate in immunoregulatory mechanisms, we investigated whether the percentage of Foxp3^+^Tregs in CD4^+ ^T cells is altered after treatment. The results are shown in Figure 3.

After the 12-week SFC therapy, the proportion of Foxp3^+^Tregs as a percentage of total CD4^+ ^T cells in the peripheral blood of COPD patients significantly increased from 3.33 ± 0.23% to 4.14 ± 0.21% (p < 0.001), as analyzed by flow cytometry. However, the percentage of Foxp3^+^Tregs in COPD patients after treatment was still significantly lower (p < 0.001) than that in the healthy subjects (6.51 ± 1.72%) (Figure 3). These data showed that the proportion of Foxp3^+^Tregs increased in COPD patients' blood after SFC inhalation.

### Relationship between the proportion of CD4^+^CD25^+^Foxp3^+ ^Treg cells and IL-17A levels in the serum of COPD patients' blood

**Figure 4:**
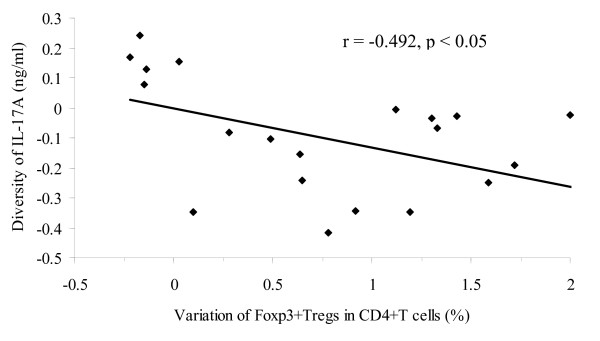
**Relationship between the IL-17A levels and the percentage of Foxp3^+^Tregs in the peripheral blood of COPD by SFC therapy**. Statistical analysis showed that there were negative correlation between the diversity of the IL-17A levels and the variation percentage of Foxp3^+^Tregs in the peripheral blood of COPD after SFC therapy (r = -0.492, p < 0.05). Correlations were determined by Spearman's rank correlation coefficients.

To investigate the relationship between the levels of IL-17A and the percentage of Foxp3^+^Tregs in the serum of COPD patients, we performed a correlation analysis of the proportion of Foxp3^+^Tregs as a percentage of total CD4^+ ^T cell number and the levels of IL-17A in peripheral blood. The results showed that, in baseline, the levels of IL-17A (1.06 ng/ml) were correlation with the proportion of Foxp3^+^Tregs in total CD4^+ ^T cells (3.33%, r = -0.783, p < 0.001, Table 5). After SFC therapy, the levels of IL-17A (0.95 ng/ml) were connect with the proportion of Foxp3^+^Tregs in total cells (4.14%, r = -0.475, p = 0.030, Table 5). Interestingly, there were the marked correlation between the diversity of IL-17A levels (-0.11 ng/ml) and the variation percentage of Foxp3^+^Tregs in CD4^+ ^T cell (0.81%) before and after SFC therapy (r = -0.492, p < 0.023, Figure 4). Obviously, statistical analysis revealed a negative correlation of IL-17A levels with the augmentation of Foxp3^+ ^Tregs in COPD patients' peripheral blood by SFC therapy.

**Table 5:** Correlation analysis on the levels of IL-17A and the percentage of Foxp3^+^Tregs in CD4^+ ^T cells in the peripheral blood of COPD by SFC therapy

	Baseline	SFC therapy	Alteration	r1	r2	r3
IL-17A (ng/ml)	1.06 ± 0.06	0.95 ± 0.06	-0.11			
Foxp3^+^Tregs (%)	3.33 ± 0.23	4.14 ± 0.21	0.81	-0.783***	-0.475*	-0.492*

r1 = -0.783, ***p < 0.001, levels of IL-17A vs. Foxp3^+ ^Tregs before SFC therapy (Baseline)

r2 = -0.475, * p < 0.05, levels of IL-17A vs. Foxp3^+^Tregs after SFC therapy

r3 = -0.492, * p < 0.05, alteration of IL-17A vs. Foxp3^+^Tregs in COPD by SFC therapy

## Discussion

TRISTAN, TORCH, and other randomized, placebo-controlled clinical trials have proven that SFC therapy for improves lung function and quality of life of patients with COPD, reduces the risk of acute exacerbation of symptoms [13,15,20,21], and decreases the rate of decline in FEV1 values [22]. In our study and other reports, SFC therapy improved pre-FEV1 values and St. George's Respiratory Questionnaire (SGRQ) scores [23], reduced the risk of acute exacerbation of COPD, reduced dyspnea according to the MMRC scale, and increased the in the 6-min walk distance. For example, Barners et al. found that SFC significantly improved pre-FEV1 values [16], and Celli et al. found that SFC decreased the rate of FEV1 decrease in COPD patients [22]. Our results and those of others indicate that long-term SFC administration can improve clinical endpoints. However, the mechanism underlying the effects of SFC therapy on COPD is unclear.

COPD is defined as an "abnormal inflammatory response" disease [24]. Therefore, development of anti-inflammatory treatments for COPD is extremely important. It has been suggested that SFC may have anti-inflammatory effects on COPD. However, there is still significant controversy regarding this subject [25]. Barnes et al. found that SFC therapy can significantly reduce the numbers of CD8^+ ^or CD4^+ ^T cells in the bronchial mucosa, but has no effect on the number of CD68^+ ^cells (monocytes/macrophages). More importantly, their study was the first to show that SFC therapy reduced the number of neutrophils in induced sputum [16]. Our findings are consistent with those of their report. Bourbeau et al. found that the numbers of CD8^+ ^cells and CD68^+ ^macrophages were reduced by treatment with SFC compared to that with a placebo, but not by treatment with FC alone. SFC therapy did not significantly change neutrophil numbers relative to placebo [26]. However, there were only 9 cases of SFC therapy in the group investigated by Bourbeau et al., which may weaken the viability of their conclusion due to the small sample size.

Our study also confirmed that SFC therapy significantly reduced the levels of two important inflammation markers, IL-8 and TNF-α, in induced sputum. IL-8 is a chemotactic factor of leukocytes and lymphocytes. Neutrophils and CD8^+ ^T lymphocytes are major inflammatory effector cells in COPD. IL-8 may activate neutrophils, thereby mediating the inflammatory response in COPD [7]. Treatment with salmeterol/FC (100/1000 μg daily) for 3 months has been reported to significantly reduce the level of IL-8 in induced sputum [25]. Meanwhile, cigarette extracts were shown to stimulate neutrophils and macrophages to release IL-8 in vitro, and this response could be inhibited by FC in a dose-dependent manner. Furthermore, the anti-inflammatory effect of FC could be enhanced by salmeterol, suggesting that salmeterol and FC have synergistic anti-inflammatory effects [27]. Similar synergistic effects of these drugs on IL-8 levels were also reported [28]. IL-8 seems to account, to some extent, for the effects of SFC on neutrophils.

TNF-α is mainly produced by monocytes and macrophages after infection. TNF-α can induce monocytes/macrophages to produce IL-8, which activates neutrophils and stimulates them to undergo chemotaxis. Activated neutrophils can release large amounts of reactive oxygen species, proteolytic enzymes, lipids, and cytokines, which further aggravate lung injury [28]. In COPD pathogenesis, TNF-α can also stimulate epithelial cells to release monocytes/macrophage- or neutrophil-derived chemotactic factors [29], leading to injury and remodeling of lung tissue. Many studies have confirmed that the degree of TNF-α elevation in the peripheral blood of COPD patients is closely related to the severity of COPD symptoms [30-32]. Baners et al. reported that SFC reduced the expression of TNF-α in the bronchial mucosa of COPD patients [16]. In addition, we found that SFC reduced the levels of TNF-α and IL-8 in the peripheral blood of COPD patients.

We also identified a significant decrease in IL-17A levels in induced sputum after SFC therapy. IL-17A, secreted by Th17 cells, induces airway epithelial cells to release IL-8 and granulocyte macrophage colony-stimulating factor (GM-CSF). GM-CSF stimulates neutrophils and macrophages to proliferate and undergo chemotaxis, and thus activates the inflammatory response in COPD. IL-17A can also induce the release of matrix metalloproteinase (MMP)-9, which is involved in emphysema [33-35]. It was previously found that the number of cells expressing IL-17A in the bronchial mucosa was significantly increased in COPD patients [36]. Our data also suggest that lower levels of IL-17A after SFC therapy may be associated with the reduced number of neutrophils in induced sputum.

More importantly, we also found that the proportion of Foxp3^+^Tregs as a percentage of total CD4^+ ^T cells in the peripheral blood of COPD patients was significantly smaller than that in the healthy subjects, and could be elevated significantly by SFC therapy. Foxp3^+ ^Tregs are a subpopulation of T cells that have anti-inflammatory and immunoregulatory effects, and thereby maintain T cell homeostasis and tolerance to self-antigens [37,38]. Correlations have been found between dysfunction of Tregs and the pathogenesis of asthma and rheumatoid and other autoimmune diseases [39,40]. Recently, Burcelo et al. found that the number of Foxp3^+^Tregs in COPD patients was significantly lower than that in smokers without COPD, but showed no difference relative to healthy subjects, which is difficult to explain. It has been found that the number of Foxp3^+^Tregs in the peripheral blood of children with asthma decreased but did not reach a significant difference compared to that in the control subjects. However, CD4^+^CD25^+ ^T cells in the lungs of these patients were significantly reduced in number. CD4^+^CD25^+ ^T cell numbers in peripheral blood can be significantly increased after treatment with inhaled corticosteroids (ICS), such as FC [41]. About the relationship between Th 17 cells (producing IL-17A) and Foxp3^+^Tregs, natural Foxp3^+^Tregs inhibited Th2 polarization and Th17-driven lung inflammation in BALB/c mice [42,43]. Anti-IL-17 treatment had significantly fewer Th1 cells and more Treg after lymph node infusion in aplastic anemia [44]. CD4^+^Foxp3^+ ^Tregs were able to control Th17 and Th17+Th1 cells in an IL-10-dependent manner in mice colitis [45]. CD39^+ ^Regulatory T cells suppress generation and differentiation of Th17 cells in human malignant pleural effusion [46].

These findings are consistent with our observations in COPD patients, suggesting that ICS treatment can increase Foxp3^+^Treg cell numbers to some extent. Foxp3^+^Tregs may also contribute to the anti-inflammatory effects of ICS/long-acting beta agonist (LABA) combination therapy. In addition, we found significant negative correlation between the number of Foxp3^+^Tregs in the peripheral blood and IL-17 levels, suggesting that Foxp3^+^Tregs may negatively regulate the differentiation of Th17 cells and IL-17 production. On the other hand, it was also reported that there was an increase in Tregs in COPD patients, his regulation of inflammation depended on the between pro- and anti- inflammatory influences [47]. Therefore, the more evidence and the connection regarding Th17 cells, IL-17 and Tregs in COPD need further study in the future.

Our study demonstrates that SFC therapy has significant anti-inflammatory effects and improves clinical symptoms of COPD. SFC administration reduced the levels of inflammation markers in sputum and peripheral blood, and reduced the number of neutrophils in induced sputum. These effects may be related to decreased levels of IL-8 and IL-17 in induced sputum and the recovery of normal Foxp3^+^Treg cell numbers in the peripheral blood. The anti-inflammatory effects of SFC may contribute to its efficacy in the treatment of COPD.

## Competing interests

The authors declare that they have no competing interests.

## Authors' contributions

GSW and CZW designed the study and the experiments. LY, QZ and QLM conducted the flow cytometry and data collection. HPC and WY performed induced sputum. LY, QLM and GSW analyzed the data. LY and GSW prepared the manuscript. CZW, QLM, WY, QZ and HPC revised and all the authors approved the final manuscript.

## Supplementary Material

Additional File 1 **Table S1.doc**. Comparison with the cell numbers in the induced sputum of COPD patients. Click here for file

## References

[B1] Rabe KF Hurd S Anzueto A Barnes PJ Buist SA Calverley P Fukuchi Y Jenkins C Rodriguez-Roisin R van Weel C Zielinski J Global strategy for the diagnosis, management and prevention of chronic obstructive pulmonary disease: GOLD executive summary Am J Respir Crit Care Med 2007 176 532 555 10.1164/rccm.200703-456SO 17507545

[B2] Hogg JC Pathophysiology of airflow limitation in chronic obstructive pulmonary disease Lancet 2004 364 709 721 10.1016/S0140-6736(04)16900-6 15325838

[B3] Rytilä P Plataki M Bucchieri F Uddin M Nong G Kinnula VL Djukanovic R Airway neutrophilia in COPD is not associated with increased neutrophil survival Eur Respir J 2006 28 1163 1169 10.1183/09031936.00149005 16971404

[B4] Cosio Manuel G Marina Saetta Alvar Agusti Immunologic Aspects of Chronic Obstructive Pulmonary Disease N Engl J Med 2009 360 2445 2454 10.1056/NEJMra0804752 19494220

[B5] Turato G Di Stefano A Maestrelli P Turato G Di Stefano A Maestrelli P Mapp CE Ruggieri MP Roggeri A Fabbri LM Saetta M Effect of smoking cessation on airway inflammation in chronic bronchitis Am J Respir Crit Care Med 1995 152 1262 1267 7551380 10.1164/ajrccm.152.4.7551380

[B6] Rutgers SR Postma DS ten Hacken NH Kauffman HF van Der Mark TW Koëter GH Timens W Ongoing airway inflammation in patients with COPD who do not currently smoke Thorax 2000 55 12 18 10.1136/thorax.55.1.12 10843943

[B7] Gadgil A Duncan SR Role of T-lymphocytes and pro-inflammatory mediators in the pathogenesis of chronic obstructive pulmonary disease Int J Chron Obstruct Pulmon Dis 2008 3 531 541 19281072 10.2147/copd.s1759 PMC2650590

[B8] Tang Q Bluestone JA The Foxp3+ regulatory T cell: a jack of all trades, master of regulation Nat Immunol 2008 9 239 44 18285775 10.1038/ni1572 PMC3075612

[B9] Jiang H Chess L An integrated view of suppressor T cell subsets in immunoregulation J Clin Invest 2004 114 1198 1208 15520848 10.1172/JCI23411 PMC524238

[B10] Lee SH Goswami S Grudo A Song LZ Bandi V Goodnight-White S Green L Hacken-Bitar J Huh J Bakaeen F Coxson HO Cogswell S Storness-Bliss C Corry DB Kheradmand F Antielastin autoimmunity in tobacco smoking-induced emphysema Nat Med 2007 13 567 569 10.1038/nm1583 17450149

[B11] Xiong XZ Jin Y Zhou Q Zhang XJ Du W Liu W Huang SA Correlation between FoxP3+ regulatory T cells and chronic obstructive pulmonary disease Zhonghua Yi Xue Za Zhi 2008 88 471 474 18642789

[B12] Welte T Optimising treatment for COPD - new strategies for combination therapy Int J Clin Pract 2009 63 1136 1149 10.1111/j.1742-1241.2009.02139.x 19624783 PMC2739483

[B13] Calverley P Pauwels R Vestbo J Jones P Pride N Gulsvik A Anderson J Maden C Combined salmeterol and fluticasone in the treatment of chronic obstructive pulmonary disease: a randomised controlled trial Lancet 2003 361 449 456 10.1016/S0140-6736(03)12459-2 12583942

[B14] Szafranski W Cukier A Ramirez A Menga G Sansores R Nahabedian S Peterson S Olsson H Efficacy and safety of budesonide/formoterol in the management of chronic obstructive pulmonary disease Eur Respir J 2003 21 74 81 10.1183/09031936.03.00031402 12570112

[B15] Mahler DA Wire P Horstman D Chang CN Yates J Fischer T Shah T Effectiveness of fluticasone propionate and salmeterol combination delivered via the Diskus device in the treatment of chronic obstructive pulmonary disease Am J Respir Crit Care Med 2002 166 1084 1091 10.1164/rccm.2112055 12379552

[B16] Barnes NC Qiu YS Pavord ID Parker D Davis PA Zhu J Johnson M Thomson NC Jeffery PK SCO30005 Study Group Antiinflammatory effects of salmeterol/fluticasone propionate in chronic obstructive lung disease Am J Respir Crit Care Med 2006 173 736 743 10.1164/rccm.200508-1321OC 16424444

[B17] Pin I Gibson PG Kolendowicz R Girgis-Gabardo A Denburg JA Hargreave FE Dolovich J Use of induced sputum cell counts to investigate airway inflammation in asthma Thorax 1992 47 25 29 10.1136/thx.47.1.25 1539140 PMC463545

[B18] Efthimiadis A Spanevello A Hamid Q Kelly MM Linden M Louis R Pizzichini MM Pizzichini E Ronchi C Van Overvel F Djukanović R Methods of sputum processing for cell counts, immunocytochemistry and in situ hybridisation Eur Respir J Suppl 2002 37 19s 23s 12361358 10.1183/09031936.02.00001902

[B19] Liu SF Chin CH Wang CC Lin MC Correlation between serum biomarkers and BODE index in patients with stable COPD Respirology 2009 14 999 1004 10.1111/j.1440-1843.2009.01608.x 19740260

[B20] Jenkins CR Jones PW Calverley PM Celli B Anderson JA Ferguson GT Yates JC Willits LR Vestbo J Efficacy of salmeterol/fluticasone propionate by GOLD stage of chronic obstructive pulmonary disease: analysis from the randomised, placebo-controlled TORCH study Respir Res 2009 10 59 19566934 10.1186/1465-9921-10-59 PMC2714501

[B21] Chung KF Salmeterol/fluticasone combination in the treatment of COPD Int J Chron Obstruct Pulmon Dis 2006 1 235 242 10.2147/copd.2006.1.3.235 18046860 PMC2707153

[B22] Celli BR Thomas NE Anderson JA Ferguson GT Jenkins CR Jones PW Vestbo J Knobil K Yates JC Calverley PM Effect of pharmacotherapy on rate of decline of lung function in chronic obstructive pulmonary disease: results from the TORCH study Am J Respir Crit Care Med 2008 178 332 338 10.1164/rccm.200712-1869OC 18511702

[B23] Zheng JP Yang L Wu YM Chen P Wen ZG Huang WJ Shi Y Wang CZ Huang SG Sun TY Wang GF Xiong SD Zhong NS The efficacy and safety of combination salmeterol (50 microg)/fluticasone propionate (500 microg) inhalation twice daily via accuhaler in Chinese patients with COPD Chest 2007 132 1756 1763 10.1378/chest.06-3009 17951625

[B24] Gómez FP Rodriguez-Roisin R Global Initiative for Chronic Obstructive Lung Disease (GOLD) guidelines for chronic obstructive pulmonary disease Curr Opin Pulm Med 2002 8 81 86 10.1097/00063198-200203000-00001 11845001

[B25] Perng DW Tao CW Su KC Tsai CC Liu LY Lee YC Anti-inflammatory effects of salmeterol/fluticasone, tiotropium/fluticasone or tiotropium in COPD Eur Respir J 2009 33 778 784 10.1183/09031936.00115308 19129278

[B26] Bourbeau J Christodoulopoulos P Maltais F Yamauchi Y Olivenstein R Hamid Q Effect of salmeterol/fluticasone propionate on airway inflammation in COPD: a randomised controlled trial Thorax 2007 62 938 943 10.1136/thx.2006.071068 17557771 PMC2117108

[B27] Mortaz E Rad MV Johnson M Raats D Nijkamp FP Folkerts G Salmeterol with fluticasone enhances the suppression of IL-8 release and increases the translocation of glucocorticoid receptor by human neutrophils stimulated with cigarette smoke J Mol Med (Berl) 2008 86 1045 1056 10.1007/s00109-008-0360-0 PMC2517086 18600309

[B28] Lindley I Aschauer H Seifert JM Lam C Brunowsky W Kownatzki E Thelen M Peveri P Dewald B von Tscharner V Synthesis and expression in Escherichia coli of the gene encoding monocyte-derived neutrophil-activating factor: biological equivalence between natural and recombinant neutrophil-activating factor Proc Natl Acad Sci USA 1988 85 9199 9203 10.1073/pnas.85.23.9199 3057503 PMC282706

[B29] Stockley RA Neutrophils and the pathogenesis of COPD Chest 2002 121 151S 155S 10.1378/chest.121.5_suppl.151S 12010844

[B30] Keatings VM Jatakanon A Worsdell YM Barnes PJ Effects of inhaled and oral glucocorticoids on inflammatory indices in asthma and COPD Am J Respir Crit Care Med 1997 155 542 548 9032192 10.1164/ajrccm.155.2.9032192

[B31] Churg A Dai J Tai H Xie C Wright JL Tumor necrosis factor-alpha is central to acute cigarette smoke-induced inflammation and connective tissue breakdown Am J Respir Crit Care Med 2002 166 849 854 10.1164/rccm.200202-097OC 12231496

[B32] Mukhopadhyay S Hoidal JR Mukherjee TK Role of TNFalpha in pulmonary pathophysiology Respir Res 2006 7 125 10.1186/1465-9921-7-125 17034639 PMC1613248

[B33] Laan M Cui ZH Hoshino H Lötvall J Sjöstrand M Gruenert DC Skoogh BE Lindén A Neutrophil recruitment by human IL-17 via C-X-C chemokine release in the airways J Immunol 1999 162 2347 2352 9973514

[B34] Jones CE Chan K Interleukin-17 stimulates the expression of interleukin-8, growth-related oncogene-alpha, and granulocyte-colony-stimulating factor by human airway epithelial cells Am J Respir Cell Mol Biol 2002 26 748 753 12034575 10.1165/ajrcmb.26.6.4757

[B35] Prause O Bozinovski S Anderson GP Lindén A Increased matrix metalloproteinase-9 concentration and activity after stimulation with interleukin-17 in mouse airways Thorax 2004 59 313 317 10.1136/thx.2003.008854 15047951 PMC1763825

[B36] Di Stefano A Caramori G Gnemmi I Contoli M Vicari C Capelli A Magno F D'Anna SE Zanini A Brun P Casolari P Chung KF Barnes PJ Papi A Adcock I Balbi B T helper type 17-related cytokine expression is increased in the bronchial mucosa of stable chronic obstructive pulmonary disease patients Clin Exp Immunol 2009 157 316 324 10.1111/j.1365-2249.2009.03965.x 19604272 PMC2730858

[B37] Bennett CL Christie J Ramsdell F Brunkow ME Ferguson PJ Whitesell L Kelly TE Saulsbury FT Chance PF Ochs HD The immune dysregulation, polyendocrinopathy, enteropathy, X-linked syndrome (IPEX) is caused by mutations of FOXP3 Nat Genet 2001 27 20 21 10.1038/83713 11137993

[B38] Fontenot JD Gavin MA Rudensky AY Foxp3 programs the development and function of CD4+CD25+ regulatory T cells Nat Immunol 2003 4 330 336 12612578 10.1038/ni904

[B39] Ehrenstein MR Evans JG Singh A Moore S Warnes G Isenberg DA Mauri C Compromised function of regulatory T cells in rheumatoid arthritis and reversal by anti-TNFalpha therapy J Exp Med 2004 200 277 285 10.1084/jem.20040165 15280421 PMC2211983

[B40] Lin YL Shieh CC Wang JY The functional insufficiency of human CD4+CD25 high T-regulatory cells in allergic asthma is subjected to TNF-alpha modulation Allergy 2008 63 67 74 18053016 10.1111/j.1398-9995.2007.01526.x

[B41] Karagiannidis C Akdis M Holopainen P Woolley NJ Hense G Rückert B Mantel PY Menz G Akdis CA Blaser K Schmidt-Weber CB Glucocorticoids upregulate FOXP3 expression and regulatory T cells in asthma J Allergy Clin Immunol 2004 114 1425 1433 10.1016/j.jaci.2004.07.014 15577848

[B42] Girtsman T Jaffar Z Ferrini M Shaw P Roberts K Natural Foxp3(+) regulatory T cells inhibit Th2 polarization but are biased toward suppression of Th17-driven lung inflammation J Leukoc Biol 2010 88 537 546 10.1189/jlb.0110044 20495073 PMC2924601

[B43] Jaffar Z Ferrini ME Girtsman TA Roberts K Antigen-specific Treg regulate Th17-mediated lung neutrophilic inflammation, B-cell recruitment and polymeric IgA and IgM levels in the airways Eur J Immunol 2009 39 3307 3314 10.1002/eji.200939498 19830731 PMC2849980

[B44] de Latour RP Visconte V Takaku T Wu C Erie AJ Sarcon AK Desierto MJ Scheinberg P Keyvanfar K Nunez O Chen J Young NS Th17 immune responses contribute to the pathophysiology of aplastic anemia Blood 2010 116 4175 4184 10.1182/blood-2010-01-266098 20733158 PMC2993623

[B45] Huber S Gagliani N Esplugues E O'Connor W Jr Huber FJ Chaudhry A Kamanaka M Kobayashi Y Booth CJ Rudensky AY Roncarolo MG Battaglia M Flavell RA Th17 cells express interleukin-10 receptor and are controlled by Foxp3^- ^and Foxp3+ regulatory CD4+ T cells in an interleukin-10-dependent manner Immunity 2011 34 554 565 10.1016/j.immuni.2011.01.020 21511184 PMC3113617

[B46] Ye ZJ Zhou Q Zhang JC Li X Wu C Qin SM Xin JB Shi HZ CD39+ regulatory T cells suppress generation and differentiation of Th17 cells in human malignant pleural effusion via a LAP-dependent mechanism Respir Res 2011 12 77 10.1186/1465-9921-12-77 21663645 PMC3120670

[B47] Lane N Robins RA Corne J Fairclough L Regulation in chronic obstructive pulmonary disease: the role of regulatory T-cells and Th17 cells Clin Sci (Lond) 2010 119 75 86 10.1042/CS20100033 20402669

