# Supplementary material for: Relationship between the anti-inflammatory properties of salmeterol/fluticasone and the expression of CD4+CD25+Foxp3+ regulatory T cells in COPD
Source: Respir Res. 2011 Oct 28;12(1):142. doi: 10.1186/1465-9921-12-142 (PMC3234191; doi:10.1186/1465-9921-12-142)
Supplement: Additional File 1 — Table S1.doc. Comparison with the cell numbers in the induced sputum of COPD patients. [file 1465-9921-12-142-S1.DOC]

**Table S1. Comparison with the cell numbers in the induced sputum** of COPD patients

|  | Baseline | | SFC therapy | | Alteration | | P value | | | | |
| --- | --- | --- | --- | --- | --- | --- | --- | --- | --- | --- | --- |
|  | Moderate  A (n=8) | Severe  B (n=13) | Moderate  C (n=8) | Severe  D (n=13) | Moderate  E (n=8) | Severe  F(n=13) | p^C-A^ | p^D-B^ | p^B-A^ | p^D-C^ | p^F-E^ |
| Total cells  (10^6^/g) | 3.10±  0.46 | 3.15±  0.44 | 2.17±  0.44 | 2.05±  0.27 | 0.93±  0.46 | 1.10±  0.39 | 0.08 | 0.02 | 0.95 | 0.80 | 0.79 |
| Neutrophils  (10^6^/g) | 2.19±  0.34 | 2.35±  0.31 | 1.23±  0.24 | 1.25±  0.18 | 0.96±  0.31 | 1.10±  0.27 | 0.02 | 0.001 | 0.74 | 0.94 | 0.74 |
| Monocytes  (10^6^/g) | 0.84±  0.16 | 0.68±  0.14 | 0.88±  0.24 | 0.72±  0.12 | 0.03±  0.27 | 0.04±  0.13 | 0.90 | 0.75 | 0.48 | 0.53 | 0.98 |
| Lymphocytes  (10^6^/g) | 0.05±  0.01 | 0.09±  0.03 | 0.04±  0.01 | 0.05±  0.01 | 0.01±  0.01 | 0.04±  0.03 | 0.25 | 0.18 | 0.22 | 0.39 | 0.45 |
| Eosinophils  (10^6^/g) | 0.02±  0.00 | 0.01±  0.00 | 0.03±  0.01 | 0.02±  0.01 | 0.01±  0.01 | 0.01±  0.02 | 0.33 | 0.43 | 0.56 | 0.80 | 0.95 |

p^C-A^: Difference of the induced sputum in moderate COPD between before (Baseline) and after SFC therapy

p^D-B^: Difference of the induced sputum in severe COPD between before (Baseline) and after SFC therapy

p^B-A^: Difference of the induced sputum between moderate and severe COPD before SFC therapy (Baseline)

p^D-C^: Difference of the induced sputum between moderate and severe COPD after SFC therapy

p^F-E^: Alteration of the induced sputum between moderate and severe COPD before and after SFC therapy
